# The cost-effectiveness of the Argus II retinal prosthesis in Retinitis Pigmentosa patients

**DOI:** 10.1186/1471-2415-14-49

**Published:** 2014-04-14

**Authors:** Anil Vaidya, Elio Borgonovi, Rod S Taylor, José-Alain Sahel, Stanislao Rizzo, Paulo Eduardo Stanga, Amit Kukreja, Peter Walter

**Affiliations:** 1 O-Zone Health Economics and Outcomes Research consultancy, Maastricht, The Netherlands; 2 Public Management & Policy Department, Bocconi University, Milan, Italy; 3 Institute of Health Research, University of Exeter Medical School, Exeter, UK; 4 Centre Hospitalier National d’Ophtalmologie des Quinze-Vingts, Paris, France; 5 Ophthalmology Department, Santa Chiara Hospital, Pisa, Italy; 6 Manchester Royal Eye Hospital, Manchester Vision Regeneration (MVR) Lab at NIHR/Wellcome Trust CRF and University of Manchester, Manchester, UK; 7 Second Sight Medical Products, Lausanne, Switzerland; 8 Department of Ophthalmology, RWTH Aachen University, Aachen, Germany

**Keywords:** Retinitis Pigmentosa, Retinal prosthesis, Cost-effectiveness analysis, Decision analytic modelling

## Abstract

**Background:**

Retinitis Pigmentosa (RP) is a hereditary genetic disease causing bilateral retinal degeneration. RP is a leading cause of blindness resulting in incurable visual impairment and drastic reduction in the Quality of life of the patients. Second Sight Medical Products Inc. developed Argus II, a retinal prosthesis system for treating RP. Argus II is the world’s first ever-commercial implant intended to restore some vision in the blind patients. The objective of this study was to assess the cost-effectiveness of the Argus® II Retinal Prosthesis System (Argus II) in Retinitis Pigmentosa (RP) patients.

**Method:**

A multi -state transition Markov model was developed to determine the cost-effectiveness of Argus II versus usual care in RP from the perspective of healthcare payer. A hypothetical cohort of 1000 RP patients aged 46 years followed up over a (lifetime) 25-year time horizon. Health outcomes were expressed as quality adjusted life years (QALYs) and direct healthcare costs expressed in 2012 €. Results are reported as incremental cost per ratios (ICERs) with outcomes and costs discounted at an annual rate of 3.5%.

**Results:**

The ICER for Argus II was €14,603/QALY. Taking into account the uncertainty in model inputs the ICER was €14,482/QALY in the probabilistic analysis. In the scenarios of an assumption of no reduction on cost across model visual acuity states or a model time horizon as short as 10 years the ICER increased to €31,890/QALY and €49,769/QALY respectively.

**Conclusion:**

This economic evaluation shows that Argus II is a cost-effective intervention compared to usual care of the RP patients. The lifetime analysis ICER for Argus II falls below the published societal willingness to pay of EuroZone countries.

## Background

Retinitis Pigmentosa (RP) is a leading cause of blindness resulting in incurable visual impairment [1]. It is a hereditary genetic disease causing bilateral retinal degeneration. It predominantly affects the photoreceptors of the retina and causes progressive loss of vision eventually leading to blindness. The prevalence of RP is estimated to be about one in 4000 affecting over one million individuals worldwide [2]. RP is usually diagnosed in young adulthood, although it can present any time from infancy to the mid-30s to 50s. Most people who have RP are legally blind by the age of 40.

Visual deficiency results in a significant economic and social disadvantage in affected individuals, their families, and society in general. Patients with a visual deficiency have more frequent medical visits, and many need assistance to perform daily life activities. RP results in a drastic reduction of the quality of life in affected individuals. In patients who have lost their sight: admission to nursing homes occurs three years earlier; the probability of falling is two times higher, the incidence of depression is three times higher; hip fractures are four times more common and the likelihood of death is twice as compared to the general population of the same age [3,4].

According to an estimate of the Age related Macular Degeneration (AMD) International Alliance, blindness and visual impairment cost the world economy nearly 2.3 trillion euros in 2010. This estimate considers the direct medical expenses for the 733 million blind or severely visually impaired people all over the world, but also the value of the time dedicated to caring for them and the loss of productivity, resulting in a loss of tax revenues that sustain the healthcare systems [5]. That means nearly 6 billion euros for the 1, 75 million affected by RP [5].

There is no treatment that can restore the functional vision or ensure regression or prevention of visual loss. Education, awareness of the disease, genetic advice and rehabilitation are used in regular practice to cope with the social and psychological impact of RP [6]. Advanced RP is associated with blindness and these patients are given independent living rehabilitation and vocational rehabilitation to promote independence and to prevent injury. Care for these patients also include formal and informal nursing care.

A retinal prosthesis placed on the retinal surface has been investigated for several years. The healthy ganglion cell layer of the retina can be stimulated by using retinal prosthesis and these implants in animal models have long-term stability [7]. Humayun et al. demonstrated the use of retinal prosthesis in human subjects [8]. Currently these retinal prostheses represent the basis for further studies towards improvement of the future devices resolution.

Second Sight Medical Products Inc. developed Argus II, a retinal prosthesis system for treating RP. Argus II is the world’s first ever-commercial implant intended to restore some vision in the blind patients. Argus II is an implantable device that works by converting video images captured from a miniature camera, housed in the patient’s glasses, into a series of small electrical impulses that are transmitted wirelessly to an array of electrodes on the surface of the retina. It has improved the visual function of patients, from minimal light perception to at least the perception of hand motions, even counting fingers. Patients can locate and recognize simple objects, see people in front of them, and follow their movement [9]. It is the world’s first and only device that has received both CE-mark and FDA approval intended to restore some functional vision for people suffering from blindness. Argus II is approved for use in the European Economic Area (CE Mark) and USA (FDA Approval).

We sought to conduct an economic evaluation of the Argus II device to inform reimbursement policy decisions and its implementation in usual practice. The aim of this study was to assess the cost-effectiveness of Argus II compared to usual care for the treatment of RP in Eurozone countries.

## Methods

The study was conducted according to the principles of good practice for decision analytic modeling according to the International Society for Pharmacoeconomics and Outcome Research (ISPOR) task force guidelines [10]. A cost utility analysis was performed from the healthcare payer’s perspective for Argus II over the time horizon (lifetime) of 25 years.

The average age of diagnosis of RP is reported to be 35.1 years and median age is 36.5 years [11]. We assumed that over a decade the visual impairment will progress to the level of legal blindness in these patients. Therefore, we simulated a hypothetical cohort of 1000 RP male and female patients aged 46 years [12].

In our model this cohort was compared to another hypothetical cohort of usual care RP patients “Care As Usual” i.e. nursing care, rehabilitation etc.

### Model structure

A Markov model with annual cycles was developed with four health states:

1. RP patients with minimal light perception, MLP

2. Visual acuity + (light perception, LP)

3. Visual acuity ++ (counting fingers, CF)

4. Visual acuity +++ (reading letters, RL)

**Figure 1:**
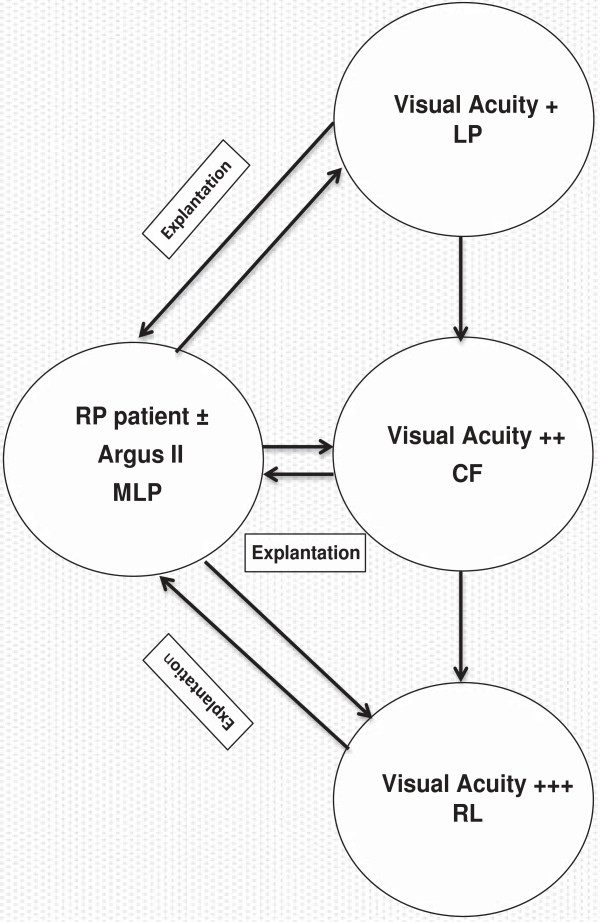
Markov model.

The model analysis began in the first state (RP patients with minimal light perception) for all the individuals. The other three Markov states represent progressive improvement in visual acuity of Argus II fitted patients. The hypothetical cohort fitted with Argus II device was followed up for the time horizon of the model for their movement into the other Markov states based on the calculated transitional probabilities. The reference hypothetical cohort with ‘Care As Usual (CAU)’ remained in the state of RP patient with minimal light perception for the entire model time horizon. The decision analytic model was developed in Microsoft excel 2010 software (Microsoft Corporation, Redmond, Washington). The schematic diagram of the Markov model is shown in the Figure 1.

### Data sources

1. Transition probabilities

A controlled, non-randomized, prospective, multi-centric study conducted in 10 sites in Europe and in the United States, showed the performance and safety of the Argus II in 30 eligible patients with severe external retinal degeneration caused by RP [13]. The results were systematically analyzed controlling the system activation (system on versus system off). These patients received the Argus II between June 2007 and August 2009. The Functional Low-Vision Observer Rated Assessment (FLORA), a functional low vision test, was developed in the year 2010 by the company Second Sight Medical Products in collaboration with a group of experts specialized in low vision rehabilitation. The ‘FLORA functional low vision test’ was completed in April 2011. Three assessment tools were utilized to assess the ARGUS II subjects namely:

a. In-depth interview of the subject by assessors

a. Observer rated task including orientation, mobility and day to day activities

a. A case study narrative written by the assessors after the assessment. This represented the totality of the assessor’s judgment and opinions about the effects of the ARGUS II system on patient’s daily life.

The data from 30 eligible RP patients implanted with the Argus II Retinal Prosthesis were used to calculate the transition probabilities for our decision analytic model. The model assumed to have Argus II fitted patients moving from low visual acuity to higher visual acuity health states at an annual rate of 10%. This transition was deemed reasonable after personal communication with experts. The Markov model transition probabilities are shown in the Table 1.

**Table 1:** Model parameters

**Name**	**Description**	**Deterministic value**	**Probabilistic components**						**Source**
			**Distribution**	**Range**		**Gamma**	**Alpha1**	**Alpha 2**	
				**Min**	**Max**				
cDR	Cost discount rate	0.035	Fixed						Pharmaco economic guidelines (NICE)
oDR	Outcome discount rate	0.035	Fixed						Pharmaco economic guidelines (NICE)
cArgus II	Costs of device + implantation	90800	BETA Pert	68100	113500	8	5	5	Second Sight
cRP	Annual health care costs incurred on RP patients	11789	BETA Pert	8841	14736	8	5	5	Frick et al. [14]
cVA+	Annual health care costs incurred on RP patients with VA+	9431	BETA Pert	7073	11789	8	5	5	Frick et al. [14] + assumption
cVA++	Annual health care costs incurred on RP patients with VA++	8252	BETA Pert	6189	10315	8	5	5	Frick et al. [14]+ assumption
cVA+++	Annual health care costs incurred on RP patients with VA+++	7073	BETA Pert	5305	8841	8	5	5	Frick et al. [14] + assumption
cSAE	Cost of management of Serious Adverse Event	1000	BETA Pert	750	1250	8	5	5	Expert opinion
cExplant	Cost of Explantation	2000	BETA Pert	1500	2500	8	5	5	Humayun et al. [9]/Second sight
cOthers	Annual other costs for Argus II patients (e.g. Upgrades etc.)	300	BETA Pert	225	375	8	5	5	Humayun et al. [9]/Second sight
pVA+	Argus II patient’s annual probability of getting VA+	0.74	BETA Pert	0.554	0.924	8	5	5	Humayun et al. [9]/Second Sight
pVA++	Argus II patient’s annual probability of getting VA++	0.21	BETA Pert	0.154	0.257	8	5	5	Humayun et al. [9]/Second sight
pVA+++	Argus II patient’s annual probability of getting VA++	0.04	BETA Pert	0.029	0.049	8	5	5	Humayun et al. [9]/Second sight
pSAE	Probability of Serious Adverse Event in Argus II patients in first year	0.3	BETA Pert	0.225	0.375	8	5	5	Humayun et al. [9]/Second Sight
pExplant	Argus II patient’s annual probability of device explantation	0.02	BETA Pert	0.013	0.021	8	5	5	Humayun et al. [9]/Second Sight
uRP	Utility value in RP patients	0.26	BETA Pert	0.195	0.325	8	5	5	Brown et al. [18]
uVA+	Utility value in RP patients with VA+	0.35	BETA Pert	0.263	0.438	8	5	5	Brown et al. [18]
uVA++	Utility value in RP patients with VA++	0.52	BETA Pert	0.39	0.65	8	5	5	Brown et al. [18]
uVA+++	Utility value in RP patients with VA+++	0.54	BETA Pert	0.405	0.675	8	5	5	Brown et al. [18]
uSAE	(dis) utility value in patients with Serious Adverse Events	0.16	BETA Pert	0.12	0.2	8	5	5	Schiffman et al. [19]
uExplant	Utility value in patients post Argus II explantation	0.26	BETA Pert	0.195	0.325	8	5	5	Assumption

### Costs

The cost of the patient screening for eligibility to Argus II implant, cost of Argus II device, cost of the surgical procedure including medications, instruments and supplies, costs of clinical follow-up and rehabilitation and cost of annual upgrade of the device were provided by the implant manufacturer Second Sight Medical Products Inc. The costs assigned to each Markov state were taken from an article published by Frick et al. [14]. In this article, annual health care cost for RP patients are published for the year 2012. Total mean annual costs per patient were estimated to be 14,988 USD in United States. These costs were converted to 2012 Eurozone costs by using purchasing power parity (PPP) from the Organization for Economic Cooperation and Development (OECD) database [15]. The total mean annual costs for RP patients in the Eurozone were estimated to be 11,789 Euros for the year 2012.

An association between the vision loss and increased risk of injury and depression is reported by Javitt et al. [16]. Excess costs for eye related and non-eye related medical care associated with blinding eye disease are also reported by the same author [16]. A reduction in RP patient’s medical and non-medical costs is expected with improved visual acuity as the result of a decrease in the frequency of falls, reduction in depression/anxiety and reduced need for home care. Cost of care in Age related Degeneration (AMD) has been reported by Hernández-Pastor et al. [17]. As there is are no robust data available regarding the reduction in the cost of care for RP patient with improving visual acuity, on the basis of Hernández-Pastor’s article we assumed a stepped reduction in cost of care (assistance from paid professionals for daily activities and social benefits received for visual disabilities for patients) according to progressive Markov states,i.e. reduction of 20% for patients with mild visual acuity improvement (light perception), 30% in patients with moderate visual acuity improvement (counting fingers) and 40% in patients with good visual acuity improvement (reading letters).

The costs of serious adverse events were 1000 Euros per event and the cost of explantation was 2000 Euros in the model. These costs are based on the actual data of serious adverse events in FLORA study participants provided by the Second Sight Medical Products Inc. These cost estimates were also confirmed by experts in the field. This economic evaluation was conducted from the payer’s perspective, so only the direct costs were included. Various cost estimates used in the model are shown in the model parameter Table 1.

### Utility values and QALYs

Health outcomes were quantified as QALYs. Argus II Retinal Prosthesis is expected to improve the quality of life by means of improving the visual acuity. Decreased depression, injury and improved functional vision and self-dependence would enhance utility for Argus II fitted RP patients. The measurement of utility values in retinal prosthesis for RP patients has not been previously undertaken. Therefore we used published utility values for the comparable patients. Brown et al. has published utility values for minimal light perception, light perception, counting fingers and reading letters. These utility values are elicited by using time trade off method in patients with ocular diseases [18]. Patients who experienced Serious Adverse Events (SAEs) after the Argus II implantation were assigned a utility reduction of 0.16 which is equivalent to the lost utility value estimated for severe dry eye in an article by Schiffman et al. [19]. In our model, after explantation of the Argus II device patients returned to the initial Markov state of ‘ minimal light perception’ and were assigned the utility value of this state i.e. 0.26. The utility values used in the model are shown in the model parameter Table 1.

### Data analysis

Cost effectiveness results are expressed as incremental cost effectiveness ratios i.e. incremental cost per QALY. We discounted costs and QALYs at an annual rate of 3.5% [20].

### Sensitivity analysis

We conducted Probabilistic Sensitivity Analysis (PSA) to account for underlying uncertainty in model inputs. Monte Carlo simulation was performed to assess the precision of cost effectiveness estimates. (in a PSA, each parameter is given a probability distribution, and uncertainty in all model parameters is then explored simultaneously using 1000 Monte Carlo simulation). In each of the iteration, Model parameters were randomly sampled across their respective distribution range. Model parameters were assigned Beta Pert probability distribution to capture the uncertainty. The BETA Pert distribution for model parameters (based on mode and ±25% range) was used as confidence intervals or standard errors were not reported in the source literature. The reported deterministic values were varied in ±25% range to calculate the minimum and maximum for BETA Pert distribution.

Net Monetary Benefit (NMB) framework expressing the net benefit of each strategy in monetary terms is applied to our cost effectiveness results [21]. A positive net monetary benefit implies that the cost of a new therapy is less than the value of the additional benefit achieved. A negative net monetary benefit implies that an intervention should be rejected, as its costs are higher than the value of the benefit achieved [22]. We have drawn Cost Effectiveness Acceptability Curves (CEACs) by plotting the proportion of the cost and effect pairs that are cost effective for a range of values of Willingness To Pay (WTP).

### Scenario analysis

The life span of Argus II Retinal Prosthesis is expected to be the lifetime of the patient. For this reason our base case model was undertaken over a 25 year time horizon. To test the cost-effectiveness of Argus II Retinal Prosthesis in alternative scenarios, the model was run for two different time horizons of 20 years and 10 years. In addition, a conservative scenario analysis was undertaken where we also ran our model in a scenario where costs were assumed to remain constant across all Markov health states, i.e. equivalent to the costs incurred by the legally blind RP patients in spite of improved visual acuity.

## Results

### Base case results

Argus II Retinal Prosthesis fitted patients incurred a discounted incremental cost of 42,455 Euros in comparison to the patient given ‘CAU’ over the 25 years of time. Progressive improvement in the visual acuity of the Argus II fitted patients resulted in gain of 2.91 incremental QALYs (discounted). Incremental cost-effectiveness ratio for Argus II was calculated to be 14,603 Euros per QALY. Base case deterministic results are presented in Table 2.

**Table 2:** Base case results

	**Deterministic**		**Probabilistic**	
**Intervention**	**Disc costs**	**Disc QALYs**	**Disc costs**	**Disc QALYs**
**Argus II**	€ 243,549	7.34	243,511	7.35
**CAU**	€ 201,094	4.44	201,493	4.44
**Increment**	€ 42,455	2.91	42,018	2.90
**ICERs**		**€ 14,603**		**€ 14,482**

### Sensitivity analysis results

**Figure 2:**
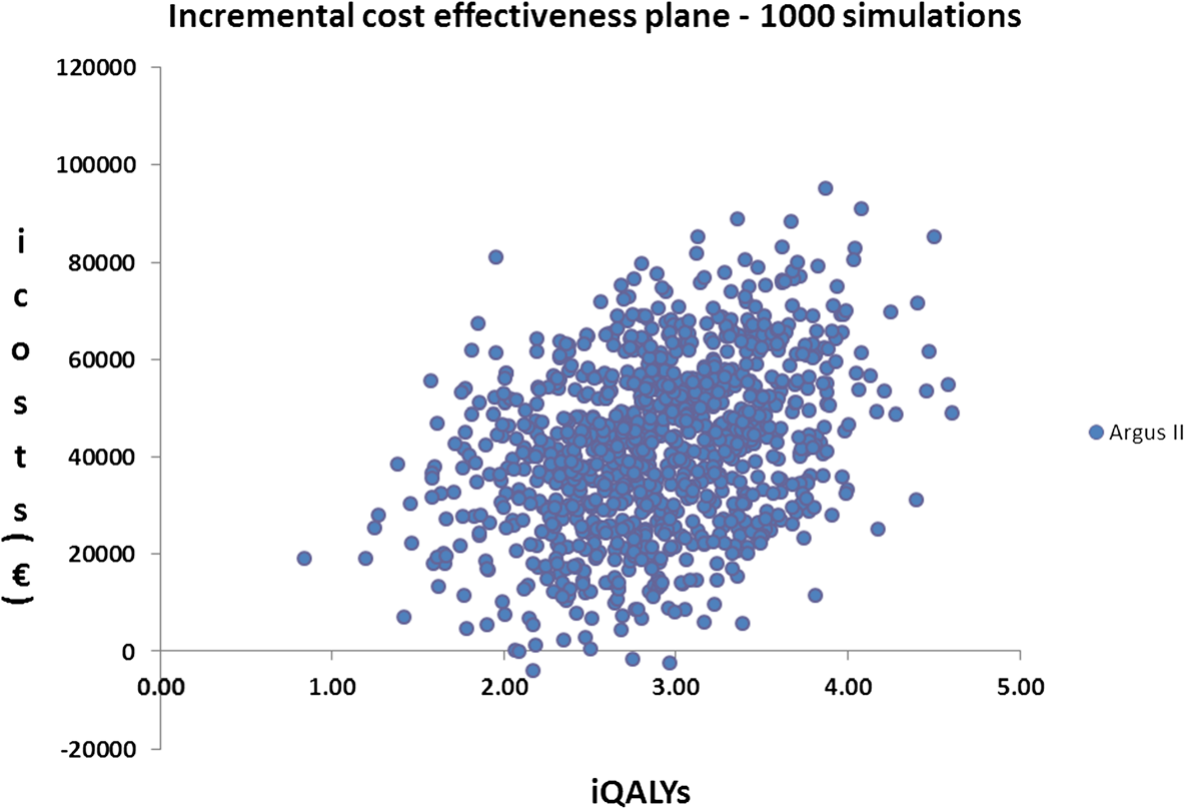
Incremental cost-effectiveness plane.

**Figure 3:**
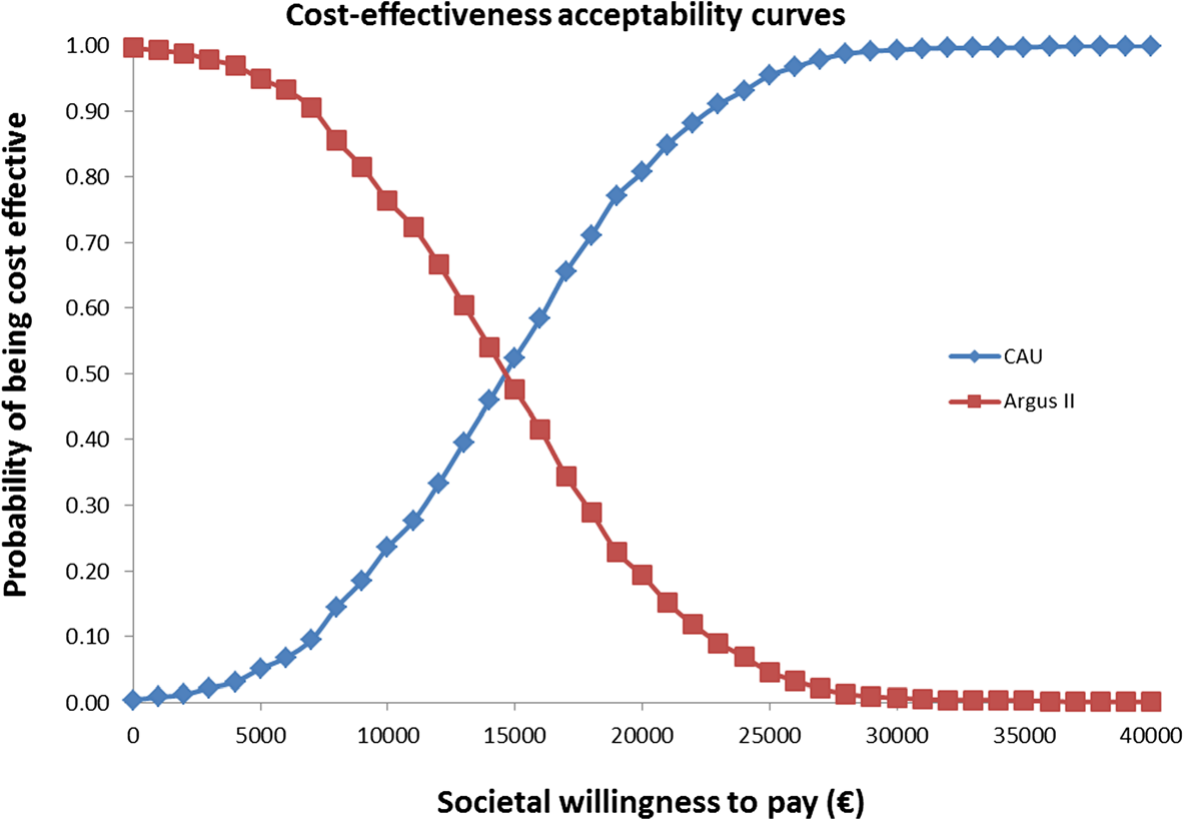
Cost-effectiveness acceptability curves (CEACs).

Probabilistic Sensitivity Analysis (PSA) showed that the cost-effectiveness of Argus II was robust to change to all key parameters within their plausible range. 1000 Monte Carlo simulations resulted in a mean incremental cost for Argus II of 42,018 Euros and mean incremental QALYs of 2.90. The Probabilistic Incremental Cost-Effectiveness Ratio (ICER) was estimated to be 14,482 Euros per QALY. The mean of incremental costs and QALY values derived from probabilistic simulations are presented in Table 2. The probabilistic model outputs are graphically illustrated in the form of incremental cost-effectiveness planes in Figure 2. In Figure 3 the CEACs show that Argus II becomes 100% cost effective at a WTP of 31000 Euros.

### Scenario analysis results

When the model was run for 20 years and 10 years Argus II fitted patients yielded 2.49 and 1.35 discounted incremental QALYs, for a discounted incremental cost of 49,128 and 67,140 Euros respectively. When costs across all health states were constant then the deterministic Incremental Cost-effectiveness Ratios (ICER) for 25 years, 20 years and 10 years’ time horizons were 31,890, 33,842 and 68,096 Euros per QALY, respectively. Probabilistic simulations also calculated ICER values very similar to the deterministic ones. The detailed results of the scenario analyses are presented in Table 3 and Table 4.

**Table 3:** Scenario analysis

**Time horizon 20 years**				
	**Deterministic**		**Probabilistic**	
			**Mean of 1000 simulations**	
**Intervention**	**Disc costs**	**Disc QALYs**	**Disc costs**	**Disc QALYs**
**Argus II**	€ 222,536	6.31	222,482	6.32
**CAU**	€ 173,408	3.82	173,752	3.83
**Increment**	€ 49,128	2.49	48,729	2.49
**ICERs**		**19,744**		**19,602**
**Time horozon 10 years**				
	**Deterministic**		**Probabilistic**	
			**Mean of 1000 simulations**	
**Intervention**	**Disc costs**	**Disc QALYs**	**Disc costs**	**Disc QALYs**
**Argus II**	€ 168,613	3.59	168,560	3.59
**CAU**	€ 101,473	2.24	101,674	2.24
**Increment**	€ 67,140	1.35	66,886	1.35
**ICERs**		**€ 49,769**		**€ 49,475**

**Table 4:** Scenario analysis

**Scenario analysis with constant costs for all Markov states (no cost reduction)**				
**Time horizon 25 years**				
	**Deterministic**		**Probabilistic**	
			**Mean of 1000 simulations**	
**Intervention**	**Disc costs**	**Disc QALYs**	**Disc costs**	**Disc QALYs**
**Argus II**	293,807	7.34	294,083	7.35
**CAU**	201,094	4.44	200,995	4.43
**Increment**	92,712	2.91	93,088	2.93
**ICERs**		**31,890**		**31,822**
**Time horizon 20 years**				
	**Deterministic**		**Probabilistic**	
			**Mean of 1000 simulations**	
**Intervention**	**Disc costs**	**Disc QALYs**	**Disc costs**	**Disc QALYs**
**Argus II**	265,906	6.31	266,216	6.32
**CAU**	173,408	3.82	173,323	3.82
**Increment**	92,498	2.49	92,893	2.50
**ICERs**		**37,174**		**37,117**
**Time horizon 10 years**				
	**Deterministic**		**Probabilistic**	
			**Mean of 1000 simulations**	
**Intervention**	**Disc costs**	**Disc QALYs**	**Disc costs**	**Disc QALYs**
**Argus II**	193,336	3.59	193,730	3.59
**CAU**	101,473	2.24	101,423	2.23
**Increment**	91,864	1.35	92,307	1.36
**ICERs**		**68,096**		**68,089**

## Discussion

Argus II is a novel healthcare technology that restores vision in RP patients. Improvements in visual acuity are expected to lead to improvements in patient self-confidence, decreasing their dependency, and to reduction in their depression/anxiety and risk of falls.

To our knowledge, this is the first formal cost-effectiveness analysis of a retinal prosthesis (Argus II) for RP patients. Compared to usual care, we found the Argus II device to be a cost effective intervention for RP patients from perspective of the healthcare payers. Over the lifetime of RP patient, the ICER for Argus II was below the published societal maximum willingness to pay thresholds of Eurozone countries.

The safety study of the Argus II Retinal Prostesis System has shown positive clinical results [13]. Since initial costs associated with the Argus II implantation may be regarded as high, it was important to conduct an economic evaluation in order to quantify the value for money of this technology in long-term health gain and costs. Cost-effectiveness analysis determines the expected impact of alternative treatment/care options. Cost-effectiveness results assist in medical decision-making by quantifying the societal benefits of a health technology against its costs and indicate treatment/care option providing the best value for money. We chose to perform the cost-utility analysis where outcome measures are QALYs. This approach allows policymaker to compare the benefits of a health technology across the health care areas due to a ‘common currency’ i.e. QALY.

Cost utility thresholds in many countries are either specified by authorities or are determined from pricing and reimbursement decisions taken in these countries. The acceptable range of this threshold in Canada is CAN$ 20,000 - 100,000 per QALY [23], in United States is US$50,000 per QALY [24], in England and Wales is £20,000-30,000 per QALY [25,26], and in The Netherlands is €20,000-80,000 per QALY [10]. Decision analytic model for the Argus II Retinal Prosthesis System predicted an ICER of 28,588 Euros per QALY for an RP patient. This lifetime ICER value is well within the range of these thresholds or societal Willingness To Pay (WTP) in the countries inside and outside the Eurozone.

This retinal prosthesis is of considerable public health interest as one of the most realistic approaches in currently untreatable retinal dystrophies. European policy towards rare diseases defines RP a rare disease: affecting less than one person in every 2000 persons. One of the objectives defined by the EU Commission’s Directorate General for Health and Consumer in article 12 of European reference network is to maximize the cost-effective use of resources particularly in the area of rare diseases [27]. Products intended for the diagnosis, prevention or treatment of a life threatening or chronically debilitating rare disease are called as orphan medicinal products. These products are often too expensive and have significant impact on patient’s health care expenditure [28]. Therefore, orphan medical products are eligible for many incentives as mentioned in the European Parliament and Council Regulation (EC) No 141/2000 of 16 December 1999 on orphan medicinal products [29]. Orphan products are likely to have higher prices for modest effectiveness and their ICERs are expected to be higher than the cost effectiveness thresholds. It has been reported that societal considerations are taken in account while evaluating orphan products as these products target medical conditions with no alternative therapy. A higher cost effectiveness threshold has been debated for products with high social value [30]. However, economic evaluation of Argus II Retinal Prosthesis System shows that it is a cost effective intervention even for conventional Willingness To Pay (WTP) thresholds. In various scenario analyses, higher ICERs for shorter time horizons are due to high initial costs of the device.

Markov model for the Argus II Retinal Prosthesis System begins at the age of 46 years as most of the RP patients are legally blind by this age. The model runs for 25 years to extrapolate associated costs and health outcomes in RP patients in view of the shorter life expectancy of visually impaired individuals. The productivity loss in RP patients is difficult to fathom. Currently it would be too optimistic to expect any change in RP patient’s productivity status after successful Argus II implantation. Realistically, in RP patients improved quality of life and self-dependence should be aimed by this device. On the other hand, Argus II is a novel technological breakthrough incurring high initial cost to the payers. Therefore, this cost-utility analysis for Argus II Retinal Prosthesis System has been performed from health care payer’s perspective. It should be noted that the Argus II subjects who participated in the clinical trial did not receive specific rehabilitation after implantation of their Argus II Retinal Prosthesis System. The analyses presented in our article were based on FLORA study data from two years of follow up of Argus II fitted patients. Based on this data an assessment of cost- effectiveness of Argus II in RP patients is suggestive of significant QALY gain for these patients. Argus II fitted patients are expected to get regular product support in terms of software updates, special training and rehabilitation etc. The company Second Sight Medical Products Inc. mandated a committee of experts in rehabilitation specialized in low vision to develop a functional rehabilitation program for both low vision and mobility. This rehabilitation program will allow Argus II fitted patients to integrate visual information in a complete way. This program starts with teaching the basic skills necessary to use the prosthesis, then identify the personal aims and objectives of the patients, and offer a combination of conventional low vision, mobility re-education, very specific for prosthetic vision, to help patients reach their objectives. This program will certainly accelerate integration of the vision prosthesis and probably increase the functional utility of the Argus II Retinal Prosthesis System for most patients. Furthermore, an improvement has been observed over the results of the clinical trial because of the refinement of the surgical procedure. At this point, it is probable that these improvements will translate into a substantial benefit. Functional utility improvement and surgical refinement would make Argus II device even more cost-effective in future economic evaluations.

A single Argus II Retinal Prosthesis System was explanted due to conjunctival erosion associated with hypotonia. The patient was successfully explanted at the 14th month postoperatively (the implant and the retinal tack) without any complication. Argus II explantation is an expensive procedure leading to the return to the minimal light perception Markov state for a RP patient. We have modeled explantation probabilities for the life time of Argus II patients based on the available data for two years. However, long term complications such as explantation cannot be predicted accurately and longer follow up data is required to model such events. The model incorporates costs for Serious Adverse Events (SAEs) in the first cycle only. As only 30% of the Argus II fitted patients had SAEs and 70% of the SAEs occurred in 3 months and 82% of the SAEs in 6 months after implantation. Similarly the utility reduction caused by SAEs is assigned to the patients experiencing the SAEs in the first year of implantation.

Strength of this analysis is its potential transferability as model inputs can be adapted to different settings. Inter- or intra-country variations in costs or patient reported outcomes can be incorporated into the model. The model’s robustness was explored in terms of uncertainty around the input parameters by varying point estimates by 25% lower and 25% higher. Running probabilistic simulations did validation of the model results. The mean of 1000 probabilistic draws revealed ICERs very similar to the deterministic values. This model conforms to the principles of good practice for decision analytic models with use of transparent data and modeling technique as per the guidelines laid by International Society for Pharmacoeconomics and Outcome Research (ISPOR) task force [10].

This study shares the general limitations of economic modeling. The analysis presented in this paper was based on the data from only 30 Argus II fitted patients followed up for 24 months. This retinal prosthesis is a novel technology that requires surgical intervention and incurs considerable costs. Data from increased numbers of Argus II fitted patients with longer follow up in the coming years provides an opportunity re-consolidate the results of our analysis. The costs and utility values for our model are taken from comparable patients. Future research should estimate costs and elicit RP patients’ preferences to determine the utility values in these patients at various visual acuity levels.

## Conclusion

This economic evaluation concludes that Argus II Retinal Prosthesis System is a cost-effective device to treat RP patients. Argus II is the only licensed device available to restore some sight in these patients. The ICER for Argus II falls below the societal willingness to pay in most EU countries. This analysis numerically demonstrates the health gain in terms of QALYs at an affordable cost in the Eurozone.

## Abbreviations

RP: Retinitia pigmentosa; QALY: Quality adjusted life years; ICER: Incremental Cost-effectiveness ratio; AMD: Age related macular degeneration; CAU: Care as usual; PSA: Probabilistic sensitivity analysis.

## Competing interests

Anil Vaidya works for O-zone Health Economics and Outcomes Research consultancy and is paid fees to conduct this research and Amit Kukrja is an employee of the Second Sight Medical Products. All other co-authors have no conflict of interest to declare.

## Authors’ contributions

Conceived and designed the economic evaluation: AV. Data: AK, PW, JAS, SR and PES. Wrote the paper: AV, AK and PW. Critical revision of the manuscript: EB, RST and PW. Final approval of the manuscript for publication: PW, EB, RST and PW. All authors read and approved the final manuscript.

## Pre-publication history

The pre-publication history for this paper can be accessed here:

http://www.biomedcentral.com/1471-2415/14/49/prepub
